# Predictors of Health Insurance Enrollment among HIV Positive Pregnant Women in Kenya: Potential for Adverse Selection and Implications for HIV Treatment and Prevention

**DOI:** 10.3390/ijerph17082892

**Published:** 2020-04-22

**Authors:** Lawrence P.O. Were, Joseph W Hogan, Omar Galárraga, Richard Wamai

**Affiliations:** 1 Department of Health Sciences, Boston University’s College of Health and Rehabilitation Sciences: Sargent College & Department of Global Health, Boston University School of Public Health, Boston, MA 02215, USA; 2 School of Public Health, Brown University, Providence, RI 02912, USA; jhogan@stat.brown.edu (J.W.H.); omar_galarraga@brown.edu (O.G.); 3 Department of Cultures, Societies and Global Studies, Northeastern University, Boston, MA 02115, USA; R.Wamai@northeastern.edu

**Keywords:** enrollment, financing, healthcare, HIV/AIDS, health insurance, Kenya, National Hospital Insurance Fund, predictors, pregnant women, socio-demographic, universal health coverage

## Abstract

*Background*: The global push to achieve the 90-90-90 targets designed to end the HIV epidemic has called for the removing of policy barriers to prevention and treatment, and ensuring financial sustainability of HIV programs. Universal health insurance is one tool that can be used to this end. In sub-Saharan Africa, where HIV prevalence and incidence remain high, the use of health insurance to provide comprehensive HIV care is limited. This study looked at the factors that best predict social health insurance enrollment among HIV positive pregnant women using data from the Academic Model Providing Access to Healthcare (AMPATH) in western Kenya. *Methods*: Cross-sectional clinical encounter data were extracted from the electronic medical records (EMR) at AMPATH. We used univariate and multivariate logistic regressions to estimate the predictors of health insurance enrollment among HIV positive pregnant women. The analysis was further stratified by HIV disease severity (based on CD4 cell count <350 and 350>) to test the possibility of differential enrollment given HIV disease state. *Results*: Approximately 7% of HIV infected women delivering at a healthcare facility had health insurance. HIV positive pregnant women who deliver at a health facility had twice the odds of enrolling in insurance [2.46 Adjusted Odds Ratio (AOR), Confidence Interval (CI) 1.24–4.87]. They were 10 times more likely to have insurance if they were lost to follow-up to HIV care during pregnancy [9.90 AOR; CI 3.42–28.67], and three times more likely to enroll if they sought care at an urban clinic [2.50 AOR; 95% CI 1.53–4.12]. Being on HIV treatment was negatively associated with health insurance enrollment [0.22 AOR; CI 0.10–0.49]. Stratifying the analysis by HIV disease severity while statistically significant did not change these results. *Conclusions*: The findings indicated that health insurance enrollment among HIV positive pregnant women was low mirroring national levels. Additionally, structural factors, such as access to institutional delivery and location of healthcare facilities, increased the likelihood of health insurance enrollment within this population. However, behavioral aspects, such as being lost to follow-up to HIV care during pregnancy and being on HIV treatment, had an ambiguous effect on insurance enrollment. This may potentially be because of adverse selection and information asymmetries. Further understanding of the relationship between insurance and HIV is needed if health insurance is to be utilized for HIV treatment and prevention in limited resource settings.

## 1. Introduction

At the core of the WHO Sustainable Development Goals (SDGs), is improving the health and well-being of the world’s people, especially the disenfranchised. Specific to health, the SDGs seek to end the epidemics of AIDS, tuberculosis, and malaria; achieve improvements in maternal and child health; and tackle the growing burden of non-communicable diseases (NCDs) [1]. One of the vehicles to achieve these health targets is Universal Health Coverage (UHC) [1]. UHC calls for access of all people to comprehensive health services at affordable costs and without financial hardship through protection against catastrophic health expenditures [2]. While great progress has been made in improving global health, the backdrop of using UHC to improve global health targets has been characterized as a ‘façade’ of an unprecedented level of global solidarity and resolve to combat persistent global health challenges [1]. 

In the HIV/AIDS space, tremendous gains have been recorded, with the number of new HIV infections and AIDS-related deaths markedly decreasing since peaking in 1997 and 2005, respectively [3]. These gains are partly the result of focusing on the HIV cascade [4,5] and mobilizing more than US $500 billion in global resources [6]. However, this progress seems to be slowing down. Reductions in new HIV infections in the past decade have slowed down, raising concerns of a resurgence, which is compounded by increasing incidences in some 50 countries [7], especially among youths and adolescents [8,9,10]. Global funding for HIV is also beginning to decline steadily [6]. According to the Kaiser Family foundation, donor spending on HIV declined by 13% from US $8.62 billion in 2014 to US $7.53 billion in 2015 [11]. The Joint United Nations Programme on HIV/AIDS (UNAIDS) estimates that US $26.2 billion will be required for a global HIV response in 2020 alone, if the efforts to end AIDS as a public health threat by 2030 are to stay on course [12]. In Kenya, a country with one of the largest HIV epidemics in the world in terms of the number of people living with HIV [13], data from 2018 shows that the overall adult HIV prevalence (~5%) and incidence (36,000 new infections annually) continues to decline, but HIV related deaths continue to plateau [14,15]. Additionally, without treatment and adherence, the risk of mother to child transmission (MTCT) of HIV among HIV positive pregnant women increases [16,17,18,19], with coverage at 74%, down from 86% [20]. While virtual elimination of MTCT is possible [21], this scenario presents the need to not only focus on access to ART and outcomes along the HIV cascade, but also to begin to institute a health systems approach focusing on financing, human resources, service delivery, and information and appropriate technology for comprehensive HIV treatment and prevention [22,23]. This would be in line with the Institute of Medicine (IOM), whose view on the future of HIV in Africa [24,25] is that health insurance does offer some promise in helping fill these health systems gaps in low and middle-income countries [26,27].

In higher income countries, health insurance has been used across the HIV treatment and prevention spectrum. In the US for example, public health insurance has facilitated access to health services beyond ART and ensured access to quality healthcare with disease progression [28,29]. Private health insurance plans are also required to provide appropriate care to those enrolled in their plans and in most cases are more effective than public insurance [30]. Additionally, health insurance has greatly influenced HIV testing, with insurance coverage increasing HIV testing rates more so among high-risk populations [31], and containing the epidemic among those without insurance [32]. In Europe, just like the US, both public resources and private health insurance offers coverage for citizens living with HIV [33,34]. However, migrants in Europe have to contend with social and structural barriers to access to care when diagnosed with HIV [35,36]. In sub-Saharan Africa, different studies have looked at the financial sustainability of HIV/AIDS and universal health coverage (UHC) programs [37], how to expand private health insurance coverage for HIV and AIDS [38], and considerations in the integration of the global HIV/AIDS response into UHC [39]. However, none of these studies has looked at the predictors of health insurance enrollment among people living with HIV (PLWH).

In Kenya, like most countries in sub-Saharan Africa (SSA), HIV programs are largely dependent on external donor funding. In 2015 for example, Kenya spent US $1.9 billion on HIV/AIDS [6]. Of that total, 15.6% was government HIV/AIDS spending, 2% was prepaid private HIV/AIDS spending, while 10.4% was out-of-pocket (OOP) payments [6]. The remaining 72% of HIV spending was from development assistance, with 59.6% going to curative care and 18.6% going to treatment and prevention [6]. The majority of the development assistance for HIV/AIDS comes from the US President’s Emergency Plan for AIDS Relief (PEPFAR) and USAID [40]. In addition, donors continue to fund majority (86.4%) of ARV needs [40]. However, the US government has signaled that it is going to reduce PEPFAR funding for Kenya from US $505 million in 2019 to US $350 million in 2020 [41]. It has also been projected that for Kenya to meet its 90-90-90 targets by 2030, it will cost an average of $525 in HIV/AIDS program costs per person living with HIV per year [37]. Given that 82% of HIV funding has come from donor assistance and OOP payments, there is an urgent and existential need to explore locally sustainable modes of HIV financing, including health insurance such as the National Hospital Insurance Fund (NHIF) [42].

Established by an Act of Parliament in 1966, the NHIF is the oldest Social Health Insurance (SHI) in sub-Saharan Africa [43] and the largest health insurer in Kenya, with private health insurance covering about 2% of the population [38]. Over the next 30 years, NHIF has instituted reforms to expand coverage. This expansion includes: the management of inpatient and outpatient schemes for government employees; the introduction of a quality improvement system; the launch of health subsidies for the poor; the revision of monthly premiums and increase in provider reimbursement rates through capitation; and the accreditation of public and private hospitals to expand the provider network [44,45,46]. Under NHIF legislation, enrollment is based on which segment of the economy one is employed in. All formal sector employees and civil servants (government employees, including military personnel) are mandatory members, and their employers are obligated to deduct their monthly premiums from a portion of their gross salary [45]. On the other hand, informal sector workers and the self-employed can join voluntarily paying a flat monthly premium [45]. While there are no co-payments or co-insurance, those not enrolled in NHIF depend on OOP payments or tax subsidized care in government facilities, where quality is questionable [46]. Specific to HIV, NHIF covers inpatient care based on health facility type, some outpatient diagnostics but not ART care. 

Nonetheless, NHIF does have the potential to be a locally sustainable source of funding for HIV care [47]. The government, through the National AIDS Control Council (NACC), is looking into how to harness the NHIF for HIV treatment and prevention [47]. One study in Kenya has looked at the impact of NHIF on obstetric outcomes for HIV positive pregnant women, showing that women have better access when they have insurance coverage [48]. Additionally, there is a dearth of studies in SSA looking at outcomes among HIV positive persons by insurance status. This study thus proposes to address this gap by looking at the predictors of health insurance enrollment among HIV positive pregnant women. Given the panacea set in the UHC agenda, understanding the drivers of health insurance enrollment within this population would be informative and critical for health insurance design, rollout, and the subsequent evaluation of health insurance’s impact in stemming HIV/AIDS. 

## 2. Methods

We use the concept of ‘Adverse Selection’ to guide our analysis. Historically, insurance has been championed as a means of equalizing the distribution and alleviation of the terrors of uncertainty due to human suffering and calamity i.e., collective protection against misfortune [49]. This should be the case, as through ‘mutual insurance, the mechanic, the laborer, and the merchant, are joined hand in hand in mutual protection against the risks of their callings; and the masses, above all, are shielded from the most blighting evil of the inequality of the human condition, and the danger of destitution [50]. Nonetheless, the promise and potential of insurance to live up to this billing has been impacted by the problems of ‘moral hazard’ and ‘adverse selection’ [51,52,53,54]. ‘Moral hazard’ refers to the change in incentives that can result from insurance protection, while ‘adverse selection’ is the theoretical tendency of low risk individuals to avoid or drop out of insurance pools, potentially leading to insurance pools containing a disproportionate percentage of high risk individuals [49,51,52,53,54]. In this analysis, we use the concept of ‘adverse selection’ to understand the determinants of health insurance enrollment among HIV positive pregnant women.

Specifically, we focus on ‘adverse selection’ given that it can suffer from the ‘dual problem’ of de-pooling health insurance pools through the same actions taken to address adverse selection [49], and its nature and structure may vary across insurance markets [55], more so in voluntary insurance markets, as is the case of NHIF in Kenya. The general response to ‘adverse selection’ has been risk classification and binding risks to insurance pools [49]. Given the ‘dual problem’ of ‘adverse selection’, an analysis of the determinants and predictors of health insurance enrollment among high risk groups, such as HIV positive pregnant women, in limited resource settings is timely and nascent. Such an analysis would help to establish the potential for the existence of ‘adverse selection’ and a probable mechanism to address the same. 

## 3. Study Setting

To analyze the determinants of health insurance enrollment among HIV positive pregnant women in Kenya, we use data from records of medical encounters within the Academic Model Providing Access to Healthcare (AMPATH) system between 2008 and 2013. AMPATH is one of the largest and most comprehensive HIV/AIDS control systems in SSA, providing care to more than 150,000 HIV positive individuals in western Kenya, testing approximately 80,000 pregnant women annually for HIV, and has robust electronic medical records (EMRs) [56,57]. AMPATH has also been at the forefront of helping the Ministry of Health (MOH) in Kenya formulate and implement healthcare policy initiatives and changes [58].

## 4. Study Design and Sample

The data used in the study is stored in the AMPATH Medical Records System (AMRS)—an electronic database of clinical encounters spanning more than 500 healthcare facilities in Western Kenya, with extensive socio-demographic, economic, clinical and biological variables [56]. The information in the AMRS is collected using encounter forms designed in a consultative process with physicians, clinical officers, and nurses in different healthcare specialties [59]. AMRS developers then program the AMRS input screens to capture the data during clinical encounters, and retrieve the information for research based on inclusion/exclusion criterion provided in study protocols [59]. The quality of the data captured is ensured using a concept dictionary that is centrally maintained and updated [60]. As such, for this analysis, the study population includes HIV positive pregnant women (ages 15–49 years), who get their HIV care at AMPATH clinics and have had a delivery (full term, preterm, miscarriage) with their NHIF enrollment status available in the dataset. Those enrolled in the NHIF are the cases and those not enrolled are the controls. From the AMRS, data programmers in the biostatistics program at AMPATH extracted information on institutional delivery and health insurance enrollment from 2008–2013. We used this de-identified EMR dataset to create a retrospective case-control cross-sectional study sample, based on the last clinical encounter (over the 5-year period) of HIV positive pregnant women with complete information on their insurance and institutional delivery status.

This research received approval from the Institutional Research and Ethics Committee (IREC) of Moi Teaching and Referral Hospital & Moi University School of Medicine in Eldoret, Kenya, with the Formal Approval Number: FAN: IREC 1090 on 10 October 2013.

## 5. Variables

The dependent variable is NHIF enrollment (Yes/No). The dataset also includes the following covariates: age, number of children, education, lost to follow-up from HIV ARV care/treatment during pregnancy, Cluster of Differentiation antigen 4 (CD4) count, travel time to clinic, and clinic site (urban or rural). This high dimensional vector of covariates allows for appropriate regression modeling in using the observed characteristics of HIV positive pregnant women to analyze the determinants of enrollment in NHIF.

## 6. Statistical Analysis

To analyze the determinants of NHIF enrollment among HIV positive pregnant women, we began by using cross-tabulation to describe the average distribution of the demographic and behavioral characteristics of HIV positive pregnant women by their NHIF status, in both the EMR and institutional delivery sample (Table 1). Next, we undertook a bivariate logistic regression analysis [61] to explore how each individual demographic, clinical and behavioral characteristic is associated with NHIF membership, the nature and strength of the association, and if it predicts enrollment in the NHIF (Table 2). In the bivariate logistic regression, the dependent variable is NHIF enrollment (Yes/No) and the predictor is a demographic, clinical or behavioral variable. Beyond the bivariate analysis, we were interested in establishing which of the demographic, clinical and behavioral characteristics, considered together, have the greatest influence on NHIF enrollment. We thus used multivariate logistic regression [62,63] to model how the different variables determine health insurance enrollment (Table 3). We implemented the multivariate regression models by using substantive theory to select the variables included in the model [64]. After model specification, we performed two goodness-of-fit (gof) tests using the ‘estat gof’ command in Stata. The first iteration was an ‘estat gof’ command with no options that performs a Pearson gof test, and the second iteration was ‘estat gof’ with the group option (we specified 10 groups) that performs a Hosmer-Lemeshow goodness-of-fit test [65,66]. The chi-square statistic from these models were 0.83 and 0.38, respectively. As such, we cannot reject our model. We also used a correlation matrix to check for collinearity [67]. The discussion of the results is based on the results from the multivariate regression model. All the analysis was done in Stata 16.

## 7. Heterogeneous Effects

Further, the drivers of health insurance enrollment may be heterogeneous for HIV positive pregnant women given their health status i.e., those who are in a worse off health state are more likely to enroll in health insurance, given their demonstrated need for access to healthcare [49]. Such differential enrollment in health insurance may be indicative of adverse selection, a crucial consideration for health insurance design and management. Previous studies that have looked at adverse selection across different insurance markets including automobile, annuities, life insurance, reverse mortgages, long-term care, crop insurance, and health insurance show that adverse selection does exist for some markets but not for others, and for some pools within similar markets and not others [55]. As such, there are conceptual reasons to expect adverse selection to vary across insurance markets and their corresponding segments [55]. For health insurance, HIV status and disease severity are likely drivers of health insurance enrollment i.e. adverse selection, and health insurers are likely to take into account HIV status and severity in adjusting for risk in their pools, as they stand to lose money [68]. This is true even in the era of ‘test and treat’, where anybody who tests positive for HIV should be put on treatment. Previous studies on the effect of test and treat have been inconsistent, showing that increasing viral suppression is achievable, but getting universal coverage is very difficult, and gaps exist between HIV acquisition and testing, and challenges exist in linking those that test positive for HIV with treatment [69,70]. For these reasons, we test for the potential for heterogeneous enrollment in the NHIF by stratifying the analysis by HIV disease severity. Additionally, in previous work in the same setting, we have shown that HIV disease severity does influence NHIF utilization patterns [48]. HIV disease severity is defined using CD4 counts with CD4 < 350 as “Severe HIV disease”, and CD4 > 350 otherwise [71]. 

## 8. Results

Table 1 shows the distribution of demographic and socio-economic characteristics of HIV positive pregnant women within the AMPATH electronic medical records system. From the EMR sample, 16% of HIV positive pregnant women are enrolled in the NHIF while in the institutional delivery sample; only 7.25% are enrolled in the NHIF, which covers institutional delivery. As shown in Table 1, in both the EMR and institutional delivery samples, the age of mothers when first pregnant at AMPATH is higher than their age at enrollment, irrespective of their health insurance status. In both samples, uninsured pregnant women have more children and a higher number of previous pregnancies. As for health seeking behavior, more HIV positive pregnant women lost to follow-up to HIV care during pregnancy are enrolled in NHIF. Moreover, the majority of the HIV positive pregnant women enrolled in the NHIF have shorter travel times to the health facility and get their care in urban clinics.

Results of the bivariate and multivariate analysis of the predictors of health insurance enrollment among HIV positive pregnant women using the confirmatory approach are presented in Table 2 and Table 3. From the bivariate analysis, HIV positive pregnant women who delivered at a health institution have three times the odds (OR 2.91; CI 1.56–5.41), of enrolling in health insurance. Those lost to follow-up to HIV care during pregnancy are four times (OR 3.77; CI 1.37–10.41) more likely to enroll in health insurance, while those enrolled in care in urban clinics are three times (OR 3.16; CI 1.98–5.05) more likely to have health insurance. The fact that currently being on ARV treatment negatively (OR 0.21; CI 0.10–0.43) predicts enrolling in the NHIF is notable, while CD4 at enrollment, and the number of days pre and post ARV initiation, does not have an effect on enrollment. The results from the multivariate analysis are similar to the univariate analysis, with being on ARV treatment still negatively associated with NHIF enrollment. Also, in the multivariate analysis, age at enrollment doubles the odds of enrolling in the NHIF (AOR 2.24; CI 1.30–3.86), and the odds of enrollment in insurance due to loss to follow-up to HIV care during pregnancy (AOR 9.90; CI 3.42–28.67), conditional on other observable characteristics almost triples in comparison to the same odds in the univariate analysis. 

### Health Insurance Enrollment by HIV Severity

To account for the potential of differential enrollment in health insurance given health status i.e., adverse selection, we stratify the analysis by HIV disease severity i.e., CD4 <350 as “Severe HIV disease,” and CD4 >350 otherwise. As shown in Table 4, HIV positive pregnant women with CD4 <350 are more likely to enroll in health insurance if they delivered at a health institution (AOR 3.69; CI 1.15–11.82) and if they are enrolled for their care in an urban clinic (AOR 5.71; CI 2.40–13.6). Additionally, for the mothers with CD4 <350, currently being on ARV treatment is negatively predictive of their enrollment in health insurance (AOR 0.03; CI 0.00–0.32), conditional on their demographic, clinical, and socioeconomic characteristics. Their age at enrollment at AMPATH is also predictive of enrollment in the NHIF (AOR 2.16; CI 0.94–4.94), but this result is marginally significant. For those HIV positive pregnant women who have CD4 >350, age at enrollment (AOR 2.92; CI 1.20–7.08) is predictive of insurance enrollment. They are also 17 times (AOR 17.42; CI 3.52–86.29) more likely to enroll in insurance if they were lost to follow-up to HIV care during pregnancy, conditional on their age, education, number of pregnancies, loss to follow-up during pregnancy HIV treatment status, and distance to and location of their HIV clinic. Being currently on ARV treatment is not a significant predictor of whether or not those with CD4 >350 enroll in health insurance. 

## 9. Discussion

This study assessed the predictors of health insurance enrollment among HIV positive pregnant women in Kenya. The analysis is informed by the rising rate of HIV related mortality and hospitalizations, as well as the need for institutional delivery for HIV positive pregnant women [3,17]. Moreover, there is a growing need for the exploration of alternative funding for HIV associated programming, given steadily declining donor funding [6,11]. Given the high-risk nature of the population analyzed, we used the concept of adverse selection [53,55] to interpret our findings. HIV positive pregnant women who deliver at a health facility had twice the odds of enrolling in insurance, were 10 times more likely to have insurance if they were lost to follow-up to HIV care during pregnancy, and three times more likely to enroll if they sought care at an urban clinic. On the other hand, being on HIV treatment was negatively associated with health insurance enrollment.

The descriptive analysis shows that insurance enrollment among HIV positive pregnant women is low. The low insurance enrollment is consistent with national level estimates that show similarly low enrollments rates (11%) among pregnant women in the general Kenyan population [43]. Being Lost to-follow-up to HIV care during pregnancy and receiving care at an urban clinic are positive predictors of insurance enrollment. Additionally, the univariate and multivariate regression analysis both indicate that, for HIV positive pregnant women, delivering at healthcare facility, being lost to-follow-up to HIV care during pregnancy, receiving care at an urban clinic conditional on other socio-demographic factors are positive predictors on enrollment in health insurance. This may be indicative of the potential for adverse selection and the existence of information asymmetries [72,73]. Low insurance enrollment among HIV positive pregnant women could be driven by insurance institutions, such as the NHIF trying to enroll lower risk patients in their insurance pool to keep the premiums, administrative, and claims costs down [49]. This attempt to have low risk pools is likely compounded by information asymmetries—one party having more information on health insurance than the other does [49]. This analysis also shows that HIV positive pregnant women getting their care in urban clinics and delivering at healthcare facilities are more likely to have NHIF. This may be the case, as these patients are likely to be privy to information that would enable them enroll in the NHIF compared to mothers delivering at home or getting their care in rural health facilities, who may have limited information [49]. Further, limited availability of accredited facilities in rural areas is due to the historical practice of the NHIF targeting facilities with in-patient capabilities, and the hierarchical structure of the system favoring higher-level urban facilities [74].

On the other hand, being on ARV treatment is negatively associated with NHIF enrollment. This interesting finding may be a signal of the ‘Lazarus effect’ and/or high-risk tolerance. The ‘Lazarus effect’ is a colloquial term used to refer to the marked improvement in HIV infected patients the longer they continue to take ART that subsequently improves their immunity [75,76]. While the reversal in frailty is welcome, it could lead to higher risk tolerance among HIV positive patients. Given the marked improvement in health, HIV positive patients might opt not to get health insurance, especially given that their access to ARV treatment is free, and the global prices of ARV have markedly declined to as low as $75 for a year’s supply of generic ARV in Africa [77]. The potential for increased access to ARV, given lower costs, is welcome, but would likely only be beneficial if adherence is high and viral suppression achieved. Otherwise, treatment failure would lead to hospitalizations associated with worse health outcomes, thus amplifying the need for health insurance. Moreover, evidence from the US shows that the use of generic HIV drugs does present barriers to desired outcomes [78]. Additionally, from a health systems perspective, increasing risk tolerance among high-risk populations would not be optimal, especially if the patients are dependent on regular uninterrupted care (ART) to maintain good health. Various studies continue to show that adherence to ART is an ongoing challenge across the world [79,80,81]. As such, continued engagement with the healthcare system—which is facilitated by health insurance—is going to be critical for HIV care, as well as achieving the global HIV targets of 90-90-90 [7].

If we take into consideration the severity of HIV, as determined by CD4 cell count, health insurance enrollment for mothers with lower CD4 is consistent with the overall results from the non-stratified sample. This may signal that, for HIV positive mothers with lower CD4 (CD4 <350), health insurance enrollment is influenced by adverse selection and information asymmetries. This should be of concern to policy makers, as this group of HIV positive mothers need better access to care, and insurance companies could ration this care in the event that they risk adjust their insurance pools [68]. Moreover, those HIV positive pregnant women with CD4 >350 are 17 times more likely to sign up for health insurance if lost to follow-up to HIV care during pregnancy. This may be because they are compensating for their sporadic encounters and may be more risky. Given the new ART treatment guidelines, this may play-out differently and, as indicated above, there is a need to undertake further analysis of adverse selection, as it manifests differently in various health insurance markets, and specific to HIV, we have a limited understanding of enrollment dynamics in developing countries [55]. Moreover, for HIV insurance risk adjustment, we need to take into consideration data and data sources, and the components of risk and financial adjustments for health insurance products [55,68].

While this analysis does show that adverse selection and information asymmetries are likely influencing health insurance enrollment among HIV positive pregnant women in Kenya, the results may be limited. First, NHIF enrollment in Kenya is largely voluntary, and may favor those in the formal sector more than those in the informal sector, as such these results may not reflect what is happening at the national level vis-a-vis health insurance and HIV. Second, the data used in the analysis is a cross-sectional sample from retrospective electronic clinical records that may not capture the full spectrum of predictors and nuances of insurance enrolment in these kinds of settings. Additionally, there may be the potential for recall bias on the capturing of health insurance status, but this would be offset by the frequency of multiple clinical encounters for each patient. Nonetheless, given the existence of limited analysis of predictors of health insurance for HIV positive populations in low and middle income countries (LMIC), the results presented here are informative for HIV healthcare systems that are similar to AMPATH in Kenya. The results can thus be used as a basis for further exploration of the relationship between HIV and health insurance [82,83]. The additional exploration can take advantage of novel longitudinal data sources that are becoming much more prevalent in the age of big data. Maximizing the full potential of these kinds of data will no doubt require resources, innovation, and the right policy frameworks and dispensation [7]. The additional exploration should also look into decision making around health insurance enrollment within low resource settings in high-risk populations, like the one studied here.

## 10. Conclusions

As global healthcare systems work towards improved HIV outcomes and sustainable financing for HIV care, it is going to be imperative to continue to explore the intersection of health insurance and access to HIV treatment and care. This analysis has shown that health insurance enrollment among HIV positive pregnant women in this setting is relatively low. However, these HIV positive pregnant women have positive odds of health insurance enrollment if they deliver at an institution, were lost to follow-up to HIV ART care during pregnancy, and were getting care at an urban clinic. On the other hand, being on HIV treatment is negatively associated with health insurance enrollment. Therefore, considerations of health insurance vis-a-vis HIV care and financing will need to take into account these factors, as they would influence the potential success of health insurance for HIV financing in limited resource settings, including the design of health insurance programs and the sub-sequent enrollment in these programs. As such, there is need for additional research to understand the nature and structure of adverse selection and information asymmetries in low/limited resource settings, combined with the use of innovation and novel data sources to address the challenges identified. Moreover, policy initiatives for increasing health insurance enrollment that align with UHC are going to be critical for sustained and enhanced HIV care and prevention moving forward toward future elimination.

## Figures and Tables

**Table 1:** Socio-Demographic Characteristics of HIV positive Pregnant Women within AMPATH EMR.

Variables	EMR Sample Mean (SD)		Institutional Delivery Sample Mean (SD)	
	NHIF	Uninsured	NHIF	Uninsured
Current Age	34.28 (5.96)	33.39 (6.39)	30.75 (4.95)	30.97 (5.55)
Age at Enrollment	29.51 (5.61)	29.03 (5.86)	26.91 (4.47)	27.42 (5.02)
Age- First Pregnant @ AMPATH	30.43 (5.92)	29.80 (6.15)	27.53 (4.74)	28.05 (5.32)
Ever Attended School (%)	97.82 (16.29)	92.17 (0.35)	98.87 (10.6)	99.30 (8.29)
Years of Schooling Completed	10.42 (3.10)	7.92 (2.92)	10.09 (2.86)	7.94 (2.82)
# of Pregnancies at Enrollment in AMPATH	2.84 (1.72)	3.47 (1.96)	2.79 (1.29)	3.37 (1.76)
# of Children	2.25 (1.64)	3.01 (1.86)	2.09 (1.43)	2.74 (1.68)
Enrolled Child at AMPATH (%)	66.42 (47.89)	68.06 (47.68)	76.4 (42.70)	79.45 (40.43)
Lost to Follow-up—During Pregnancy (%)	1.71 (16.67)	1.00 (15.06)	5.62 (23.16)	1.55 (12.38)
Currently on ARV Treatment	86.94 (34.76)	87.32 (33.28)	88.76 (31.76)	97.50 (15.63)
CD4 @ Enrollment	338.24 (247.14)	359.24 (307.48)	410.40 (255.90)	396.49 (327.03)
Travel Time (Hours)	1.97 (0.99)	2.05 (0.98)	1.89 (0.94)	2.00 (0.95)
# Days Pre ARV Initiation	286.46 (475.11)	271.15 (429.65)	262.07 (354.62)	227.13 (354.62)
# Days Post ARV Initiation	1828.68 (876.28)	1635.62 (784.12)	1535.34 (952.05)	1427.11 (768.71)
Urban Clinic (%)	70.06 (47.59)	44.80 (50.0)	69.70 (46.23)	42.06 (49.39)
Observations	1997 (15.86%)	10596 (84.14%)	89 (7.25%)	1159 (92.75%)
Overall N	12,593		1248	

Notes: Descriptive characteristics of HIV positive women in the AMPATH program. (SD = standard deviations, # = number).

**Table 2:** Predictors of Health Insurance Enrollment among HIV positive Pregnant Women.

Predictors	Bivariate Analysis		
	Odds Ratio	95% CI	*p*-Value
Delivered at Health Institution	2.91 (0.92)	1.56–5.41	0.00 ***
Current Age	0.99 (0.02)	0.96–1.03	0.67
Age at Enrollment	0.98 (0.02)	0.94–1.02	0.02 **
Age at First Pregnancy at AMPATH	0.98 (0.02)	0.95–1.02	0.32
Ever Attended School	0.61 (0.65)	0.08–4.95	0.65
Years of Schooling Completed	1.32 (0.06)	1.22–1.44	0.00 ***
# of Pregnancies at Enrollment in AMPATH	0.80 (0.05)	0.71–0.91	0.00 ***
Number of Children	0.76 (0.06)	0.65–0.88	0.00 ***
Enrolled Child in AMPATH	0.84 (0.22)	0.50–1.40	0.50
Lost to Follow-up—During Pregnancy	3.77 (1.95)	1.37–10.41	0.01 **
Currently on ARV Treatment	0.21 (0.08)	0.10–0.43	0.00 ***
CD4 at Enrollment	1.00 (0.00)	0.99–1.00	0.61
# Days Pre ARV Initiation	1.00 (0.00)	0.99–1.00	0.33
# Days Post ARV Initiation	1.00 (0.00)	0.99–1.00	0.29
Travel Time (Hours)	0.89 (0.11)	0.69–1.12	0.29
Enrolled in an Urban Clinic	3.16 (0.75)	1.98–5.05	0.00 ***

Notes: In parentheses are the standard deviations. # denotes “number”. Significance levels: *** *p* < 0.01, ** *p* < 0.05.

**Table 3:** Predictors of Health Insurance Enrollment among HIV positive Pregnant Women.

Predictors	Multivariate Analysis		
	Odds Ratio	95% CI	*p*-Value
Delivered at Health Institution	2.46 (0.86)	1.24–4.87	0.01 **
Current Age	0.52 (0.13)	0.31–0.86	0.01 **
Age at Enrollment	2.24 (0.62)	1.30–3.86	0.00 ***
Age at First Pregnancy at AMPATH	0.86 (0.08)	0.72–1.02	0.09 *
Ever Attended School	0.10 (0.12)	0.01–0.91	0.04 **
Years of Schooling Completed	1.28 (0.06)	1.16–1.40	0.00 ***
# of Pregnancies at Enrollment in AMPATH	0.91 (0.14)	0.68–1.22	0.54
Number of Children	1.01 (0.19)	0.70–1.45	0.98
Enrolled Child in AMPATH	0.78 (0.24)	0.43–1.41	0.40
Lost to Follow-up – During Pregnancy	9.90 (5.37)	3.42–28.67	0.00 ***
Currently on ARV Treatment	0.22 (0.09)	0.10–0.49	0.00 ***
CD4 at Enrollment	1.00 (0.00)	0.99–1.00	0.41
# Days Pre ARV Initiation	1.00 (0.00)	1.00–1.00	0.00 ***
# Days Post ARV Initiation	1.00 (0.00)	1.00–1.00	0.00 ***
Travel Time (Hours)	1.02 (0.14)	0.79–1.33	0.87
Enrolled in an Urban Clinic	2.50 (0.63)	1.53–4.12	0.00 ***

Notes: In parentheses are the standard deviations. # denotes “number”. Significance levels: *** *p* < 0.01, ** *p* < 0.05, * *p* < 0.1.

**Table 4:** Predictors of Insurance Enrollment among HIV positive Pregnant Women: By HIV Disease Severity.

Predictors	CD4 ≤ 350			CD4 > 350		
	Odds Ratio	95% CI	*p*-Value	Odds Ratio	95% CI	*p*-Value
Delivered at Health Institution	3.69	1.15–11.82	0.03 **	1.82	0.75–4.45	0.19
Current Age	0.49	0.22–1.08	0.08 *	0.50	0.25–1.02	0.06 *
Age at Enrollment	2.16	0.94–4.94	0.07 *	2.92	1.20–7.08	0.02 **
Age at First Pregnancy at AMPATH	0.92	0.75–1.13	0.41	0.70	0.48–1.01	0.06 *
Ever Attended School	1.00	0.56–1.32	0.89	0.02	0.00–0.33	0.01 **
Years of Schooling Completed	1.10	0.97–1.25	0.13	1.48	1.28–1.71	0.00 ***
# of Pregnancies at Enrollment in AMPATH	1.00	0.65–1.53	0.98	0.83	0.53–1.29	0.41
Number of Children	1.03	0.62–1.73	0.91	1.02	0.57–1.82	0.96
Enrolled Child in AMPATH	0.65	0.26–1.62	0.36	0.91	0.37–2.23	0.83
Lost to Follow-up—During Pregnancy	3.06	0.61–15.18	0.17	17.42	3.52–86.29	0.00 ***
Currently on ARV Treatment	0.03	0.00–0.32	0.00 ***	0.48	0.17–1.38	0.18
# Days Pre ARV Initiation	1.00	0.99–1.00	0.12	1.00	1.00–1.00	0.01 **
# Days Post ARV Initiation	1.00	1.00–1.01	0.03 **	1.00	1.00–1.01	0.02 **
Travel Time (Hours)	1.24	0.80–1.93	0.33	0.84	0.60–1.17	0.30
Enrolled in an Urban Clinic	5.71	2.40–13.6	0.00 ***	1.19	0.61–2.31	0.62
*N* (Sample Size)	640			607		

Notes: # denotes “number”. Significance levels: *** *p* < 0.01, ** *p* < 0.05, * *p* < 0.1.
